# Targeting Lrig1 on the surface of Treg cell in the periphery alleviates the symptoms of Alzheimer’s Disease in vivo

**DOI:** 10.1002/alz.091984

**Published:** 2025-01-09

**Authors:** Sang‐Kyou Lee

**Affiliations:** ^1^ Good T Cells, Seoul, Mapo‐gu, Korea, Republic of (South); YONSEI University, Seoul, Seodaemun‐gu Korea, Republic of (South)

## Abstract

**Background:**

Neurodegenerative diseases, including Alzheimer’s disease (AD), have been long thought to be independent of the peripheral immune system, but their pathogenesis status is functionally influenced by various T cell subsets in the periphery. Especially Treg cells are emerging as an important dynamic population in the brain, but the detailed immunological molecular and cellular processes are poorly characterized

**Method:**

We reported that the cell surface protein Lrig1 is enriched in Treg cells and is an essential regulator of the functions of Treg cells in vitro and in vivo. To evaluate the functional importance of Treg cells in AD pathogenesis, the modulating mAb specific to Lrig1 (GTC 310‐01) via intravenous injection route was administered into 5xFAD or 6xTg mice, the genetic mouse model of AD, and the various AD symptoms were investigated.

**Result:**

GTC 310‐01 significantly improved the cognitive ability of the AD mice, which were analyzed using three different methods (Y‐maze test, passive avoidance test and NOR test). And the level of Ab42 and tau aggregates in AD mice’s hippocampus and cortex areas were substantially reduced by GTC 310‐01 treatment. When the immunological and functional status of T cell subsets in the periphery of the treated animal was evaluated, GTC 310‐01 treatment inhibited the functions of Treg cell. It enhanced gIFN secretion, which increased the permeability of choroid plexus and other BBBs. Thereby, the monocytes in the periphery migrated into the brain and differentiated into active microglial cells and macrophages responsible for clearing the Ab42 and tau aggregates in the brain. After the initial pro‐inflammatory phase after GTC 310‐01 treatment, the number of Treg cells in the brain increased and created the anti‐inflammatory environment to resolve the inflammatory insults.

**Conclusion:**

In conclusion, functional modulation of Treg cells using GTC 310‐01 mAb specific to Lrig1 in the periphery could be a novel therapeutics for treating AD conditions without BBB penetration.

